# UV Laser‐Induced Carbon Microelectrode Arrays for Neuronal Recordings

**DOI:** 10.1002/adhm.202502136

**Published:** 2025-08-20

**Authors:** Fulvia Del Duca, Koji Sakai, Beatrice De Chiara, Toichiro Goto, Defne Tüzün, Lukas Hiendlmeier, George Al Boustani, Hu Peng, Tetsuhiko F. Teshima, Simon N. Jacob, Bernhard Wolfrum

**Affiliations:** ^1^ Neuroelectronics Munich Institute of Biomedical Engineering Department of Electrical Engineering School of Computation Information and Technology Technical University of Munich Hans‐Piloty‐Str. 1 85748 Garching Germany; ^2^ Basic Research Laboratories NTT, Inc. 3‐1 Morinosato Wakamiya Atsugi Kanagawa 243‐0198 Japan; ^3^ NTT Bio‐Medical Informatics Research Center NTT, Inc. 3‐1 Morinosato Wakamiya Atsugi Kanagawa 243‐0198 Japan; ^4^ Medical and Health Informatics Laboratories NTT Research Incorporated 940 Stewart Dr Sunnyvale CA 94085 USA; ^5^ Translational Neurotechnology Laboratory Department of Neurosurgery Klinikum rechts der Isar Technical University of Munich 81675 Munich Germany

**Keywords:** laser‐induced carbons, multi‐electrode arrays, neuronal recordings, parylene, UV lasers

## Abstract

Bioelectronic devices for in vitro and in vivo studies benefit from polymeric materials as substrates and insulations due to their flexible nature. Laser‐induced carbon formation has emerged as a rapid and versatile technique to fabricate conductive carbon‐based structures from insulating polymer films. Here, the development of electrodes fabricated via ultraviolet (UV) laser‐induced carbonization of chlorinated poly‐p‐xylylene (parylene‐C) insulation areas is reported. The parylene‐derived carbon is directly fabricated over the thin metallization layer, thus opening the desired electrode areas in a one‐step process. The optimal laser parameters for electrode performance are investigated, and the stability of the electrodes is tested under 10 000 voltage‐controlled stimulation pulses. In vitro tests of primary neuronal cultures confirm the biocompatibility of the proposed interfaces and reveal the good conformability of the neurons over the rough carbon structures. The performance of the sensor arrays is shown in electrophysiological recordings of neuronal cultures, together with a proof‐of‐principle stimulation, confirming the stability of the recordings over at least 4 weeks in culture. The proposed laser‐induced carbon electrodes from polymer coating are suitable as a rapid and precise fabrication protocol for carbon‐based sensors, applicable to bioelectronics and neuroelectronics devices.

## Graphical abstract

Thin‐film electrodes are flexible devices of high conformability and minimal dimensions with attractive properties for in vivo and in vitro bioelectronics. Here, electrodes are developed from parylene films via ultraviolet laser‐induced carbonization. The performance of the carbon electrodes in recording and stimulating primary neuronal cultures is tested, and the versatility of the fabrication in various designs is shown.

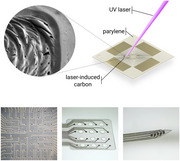

## 1 Introduction

Bioelectronics is an expanding research field that focuses on the connection between living organisms and electronics for many applications, such as monitoring,^[^ ^1^, ^2^, ^3^ ^]^ diagnostics,^[^ ^4^, ^5^ ^]^ therapy and electroceuticals,^[^ ^6^, ^7^, ^8^, ^9^, ^10^ ^]^ drug development,^[^ ^11^, ^12^, ^13^, ^14^ ^]^ and wearables.^[^ ^15^, ^16^, ^17^, ^18^, ^19^, ^20^ ^]^ Bioelectronic devices traditionally rely on silicon substrates and on metallic components for conductive tracks and for sensing or stimulation sites.^[^ ^21^, ^22^ ^]^ Silicon technology has pioneered the field of implantable neural interfaces, and has enabled advances such as high‐density recordings or multimodal integration with optogenetics and drug delivery systems.^[^ ^22^, ^23^, ^24^ ^]^ Since the last decade, polymeric materials have been exploited as substrates and coatings of bioelectronic devices for their reduced thickness and for their increased flexibility, thus better conforming to soft and dynamic biological environments.^[^ ^25^, ^26^, ^27^, ^28^, ^29^, ^30^ ^]^ However, flexible and soft bioelectronics present several challenges. First, their fabrication by conventional electronics manufacturing methods is often difficult or incompatible with standard clean‐room processing techniques.^[^ ^31^ ^]^ Additionally, the insufficient long‐term stability of the polymer–metal interface at the open electrode areas is frequently reported, and often identified as a leading cause for device failure.^[^ ^32^, ^33^, ^34^ ^]^ For example, in the first study of explanted polyimide electrodes tested for intrafascicular nerve stimulation, Čvančara et al. found that 62.5% of electrodes experienced delamination of the metallization from the polyimide–platinum interface after 30 days in vivo.^[^ ^35^ ^]^

Carbon‐based conductive materials have been proposed as an alternative or added coating to the metallization layer.^[^ ^36^, ^37^ ^]^ Carbon presents several advantages for bioelectronics as an electrode material. First, carbon‐based materials are considered superior to many other electrode materials for their biocompatibility and their high chemical inertness.^[^ ^38^, ^39^ ^]^ Additionally, carbon‐based materials exhibit an adequate electrical conductivity – albeit generally lower than that of noble metals – and a high charge injection capacity. In particular, carbon‐based materials such as carbon nanotubes (CNTs) or poly(3,4‐ethylenedioxythiophene)–polystyrene sulfonate acid (PEDOT: PSS) are valuable electrode coatings to achieve a lower impedance.^[^ ^40^, ^41^ ^]^ Finally, thanks to the wide potential window, carbon‐based materials have demonstrated good selectivity and sensitivity for electrochemical detection, and have been used in sensors for the detection of neurotransmitters and other electrically active biomolecules.^[^ ^42^, ^43^, ^44^, ^45^ ^]^

Carbon has been synthetically prepared from precursors using high‐temperature furnaces.^[^ ^46^ ^]^ For example, carbon electrodes can be fabricated through oven pyrolysis of SU‐8 photoresist, which can be photolithographically patterned in 2D or 3D structures prior to thermal treatment.^[^ ^47^, ^48^, ^49^ ^]^ In the last decade, thermochemical processes induced by lasers have proven to be a great alternative to traditional heat processes for carbon synthesis.^[^ ^50^ ^]^ In particular, laser‐induced carbonization has been investigated extensively since its rediscovery in 2014, as a rapid fabrication of carbon‐based electrodes, particularly on flexible or thin‐film polymer substrates.^[^ ^51^, ^52^ ^]^ Laser‐induced carbon, also known as laser‐induced graphene (LIG) depending on its graphene content, is a class of 3D porous carbon‐based materials obtained by converting a carbon‐rich insulating precursor into a conductive layer via laser irradiation.^[^ ^53^ ^]^ The resulting carbon differs from both glassy and activated carbons owing to the fact that this process happens within a short time, and the breaking of chemical bonds is rapid.^[^ ^54^ ^]^

The family of LIG materials has gained significant attention in the last decade due to its many advantages and its applicability in a variety of different fields. The highly porous structure due to the rapid release of volatile components during laser irradiation is advantageous for gas sensing, electrochemical biosensing, strain sensing, and energy storage applications, for example.^[^ ^55^, ^56^, ^57^, ^58^, ^59^, ^60^ ^]^ LIG has also been shown to have adequate electrical conductivity and biocompatibility, thus being a promising material for bioelectronics.^[^ ^61^, ^62^, ^63^, ^64^ ^]^ The rapidity of the one‐step laser fabrication and the tunability of the parameters make this process applicable to several carbon‐rich precursors, ranging from synthetic materials^[^ ^65^ ^]^ to cellulose and wood,^[^ ^53^, ^66^ ^]^ textiles,^[^ ^67^ ^]^ or even bread.^[^ ^68^ ^]^ Laser‐induced carbon materials from plastic materials have shown the best resulting conductivities, due to the reduced impurities and appropriate thermal properties. Specifically, polyimide (PI) is most often the material of choice due to its high heat resistance.^[^ ^51^ ^]^

Another interesting base material as substrate and insulating layer for bioelectronic devices is parylene‐C, due to its flexibility, biostability, and biocompatibility. It offers a distinct advantage over many other polymers through its deposition process, which utilizes chemical vapor deposition. This method takes place in a dry environment at room temperature, resulting in a uniform, conformal insulation film that precisely coats all exposed 3D structures. Thus, direct integration of electrode structures in parylene‐C films provides a versatile method for fabricating flexible electrode arrays for bioelectronics. Carbon electrodes from parylene‐C have been obtained from infrared radiation (IR) and characterized by Correia et al., achieving sheet resistances as low as 9.4 Ω sq^−1^ and demonstrating the feasibility of creating highly conductive graphene structures on parylene substrates.^[^ ^69^ ^]^

Early work by Gross et al. already demonstrated the potential of ultraviolet (UV) laser processing for polymer modification in microelectrode fabrication,^[^ ^70^, ^71^ ^]^ using a pulsed nitrogen laser to locally ablate polymer coatings with high focus precision. More recently, UV lasers have been used in the direct formation of laser‐induced carbon from PI.^[^ ^72^, ^73^ ^]^ In principle, UV lasers can also be used for carbon formation on parylene substrates, since UV radiation over 250 nm interacts with parylene films impacting their thermal stability and electrical properties.^[^ ^74^ ^]^

Besides being an interesting and versatile material on its own, laser‐induced carbon has the potential to be easily integrated with many existing processes and devices. The idea of combining the advantages of laser‐induced carbon with the high‐density design achievable with metal circuitry was proposed by Lu et al.,^[^ ^75^ ^]^ where a CO_2_ laser induces porous graphene electrode sites, and the metal layer is subsequently deposited and patterned. Vomero et al. have demonstrated that the inverse order of fabrication is also possible, namely that an infrared CO_2_ nanosecond laser can rapidly and locally carbonize polymers over metal components, in the case of a 25‐µm‐thick platinum/iridium foil, obtaining electrodes with diameters of 200 µm or larger.^[^ ^76^, ^77^ ^]^

Here, we show that a UV nanosecond laser can be used to obtain a laser‐induced carbon coating smaller than 100 × 100 µm^2^ on a microelectrode array (MEA) with 100‐nm‐thin gold conductor traces coated with 5 µm of parylene‐C. Further, we show the interaction between the rough carbon structure and primary neuronal cultures, as well as the stable long‐term recording and stimulation capabilities. The proposed carbon electrodes fabricated directly over thin metal layers in a one‐step process can be easily integrated in various established designs and applications in bioelectronics, from rigid MEA substrates to free‐standing implantable thin‐film devices. Future work will focus on implementing the electrodes in higher‐density designs for thin implantable neural sensors.

## 2 Results and Discussion

### 2.1 Device Fabrication

**Figure 1:**
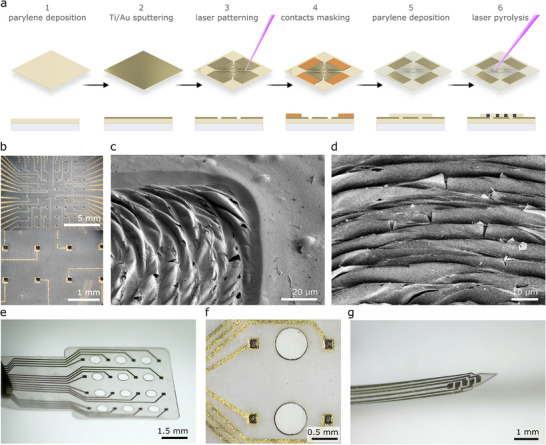
Fabrication and resulting electrodes. a) Schematic illustration of the fabrication steps. b) Optical microscopy images of the fabricated MEA. c,d) SEM images of the laser‐induced carbon electrodes. e–g) Optical microscopy images of electrode arrays of various designs for thin‐film neural interfaces: e,f) ECoG electrode array, and g) intracortical electrode array.

**Figure** **1**a shows the steps for fabricating the laser‐induced carbon electrodes studied here. First, a 5 µm layer of parylene‐C is deposited via chemical vapor deposition. To manufacture the conductor traces, thin layers of 15 nm Ti (acting as an adhesion promoter) and 100 nm Au are sputtered, and patterned via laser ablation. The contact pads are masked with Kapton tape, and a second 5 µm parylene‐C layer is deposited.

Last, the electrode openings are obtained via inducing carbonization of the carbon‐rich precursor parylene‐C over the thin metal layer. The maximum resolution of the metal tracks that can be obtained by laser micromachining is a function of the laser's beam focus and the processed material. For the UV nanosecond pulsed laser used here, it was demonstrated that feedline widths down to at least 30 µm can be reached^[^ ^78^ ^]^ thus making this process in principle translatable to devices with narrower dimension requirements such as bioelectronic or neural implants.

Similarly, the minimum size of the carbon electrode obtained by laser pyrolysis is dependent on the laser spot size, in addition to the laser parameters directly affecting the thermochemical mechanisms occurring in the irradiated area. At long‐wavelength excitations, the conversion is believed to be primarily induced by photothermal effects.^[^ ^51^ ^]^ At the shorter wavelengths of the UV range, direct photochemical processes are also probable.^[^ ^79^, ^80^ ^]^ Secondary products are thus released, influencing structure and material properties. Photothermal effects are typically also present in UV ablation processes, including thermal decomposition. The energy not utilized for bond breaking is transferred to the material matrix, resulting in a temperature increase.^[^ ^81^ ^]^ For an in‐depth description of the laser carbonization process of polymers, the reader is directed elsewhere.^[^ ^50^ ^]^

We can observe the 3D nature of the resulting carbon structure from the scanning electron microscopy (SEM) images in Figure 1c,d. The parylene in the irradiated area is converted into a carbon‐based, ridge‐like coating over the underlying Au layer. Due to the abrupt transformation of the top parylene layer, mechanical and thermal stresses are generated at the polymer/metal interface, leading to local deformation of the thin metal layer (Figure S1, Supporting Information). The modalities of heat dissipation during carbonization critically affect the damage caused to the metal. In the present approach, the underlying metal layer is not significantly damaged by UV irradiation, as the residual heat is dissipated well by the glass carrier. We have observed that the same laser‐induced process applied to free‐standing parylene/Au/parylene stacks already detached from the glass carrier (as opposed to the present approach) results in critical damage to the gold layer in the irradiated area. Different carrier materials can thus be investigated in a future study to further optimize the properties and the dimensions of the induced carbon structures. Additionally, irradiation setups with controlled substrate temperature might be a further point of investigation. Figure 1e–g shows the suitability of the approach also for the manufacturing of thin‐film electrodes of various designs, for example, for the use as electrocorticography (ECoG) or intracortical neural interfaces, by releasing the electrodes from the glass substrates.

**Figure 2:**
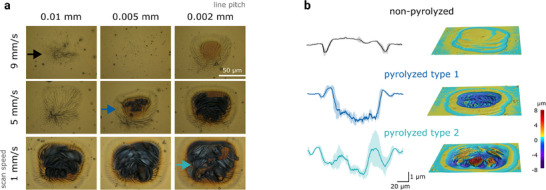
Laser pyrolyzed electrode areas. a) Optical microscopy images of the electrodes at 3 different conditions (non‐pyrolyzed, pyrolyzed type 1, pyrolyzed type 2). Two laser parameters (scan speed and line pitch) are varied as shown (common scanning parameters: 400 kHz frequency, 0.2% power, 4 mm defocus, 1 repetition). b) Laser microscopy images of 3 example areas corresponding to the laser scanning conditions in (a) (left: average profile of *n* = 3 samples per condition, mean ± SD; right: 3D images).

**Figure** **2**a shows optical microscopy images of 50 × 50 µm^2^ electrode areas obtained with different laser parameters. At higher scan speeds (9 mm s^−1^) and wider scan line pitches (0.01 mm), the laser irradiation on the parylene layer was not sufficient to induce visible carbonization (non‐pyrolyzed, Figure 2b). By reducing the speed, the overlap between subsequent laser pulses on the same parylene spot increased enabling the accumulation of more energy and promoting the carbonization process. Decreasing the line pitch had a similar result in promoting parylene carbonization. For example, at 5 mm s^−1^ speed and 0.005 mm pitch, sufficient energy converted the parylene insulation layer to the observable carbonized area (pyrolyzed type 1). When combining slower scan speeds with finer line pitches (e.g., at 1 mm s^−1^ speed and 0.002 mm pitch, pyrolyzed type 2), the conversion of parylene into a carbon coating became more effective.

We verified the effect of laser parameters by measuring the impedance at 1 kHz for different laser parameters, and the lowest impedance was found forelectrodes fabricated at 1 mm s^−1^ speed and 0.002 mm pitch (Figure S2, Supporting Information). We analyzed the resulting structures from the three exemplary conditions (non‐pyrolyzed, pyrolyzed type 1, pyrolyzed type 2) via 3D laser microscopy, as shown in Figure 2b. With large line pitch and fast scan speed, the parylene area remains largely unchanged, with only the edges of the scanned area showing an indentation of ≈1 µm depth (non‐pyrolyzed). With increasing energy, the shrinkage of the parylene layer into a carbonized coating leads to an indentation of the scanned area ≈2–2.5 µm deep (pyrolyzed type 1). This is likely due to the thermochemical decomposition of the carbon precursor.^[^ ^82^, ^83^ ^]^ At the highest line density and the lowest scan speed, we do not only observe a similarly deep indentation in the parylene layer, but also a protruding carbonaceous structure as high as 2–4 µm (pyrolyzed type 2). The formed 3D ridges can be attributed to the release of gaseous material occurring during the conversion process, which confers the typical microporous quality of the laser‐induced carbons.^[^ ^52^ ^]^ The effective conversion of parylene‐C coating to carbon coating, due to its porous nature, also leads to an increased carbonized area compared to the nominal area. For a nominal resolution of 50 × 50 µm^2^, we obtain an optically observable area of ≈90 × 70 µm^2^.

### 2.2 Electrode Characterization

**Figure 3:**
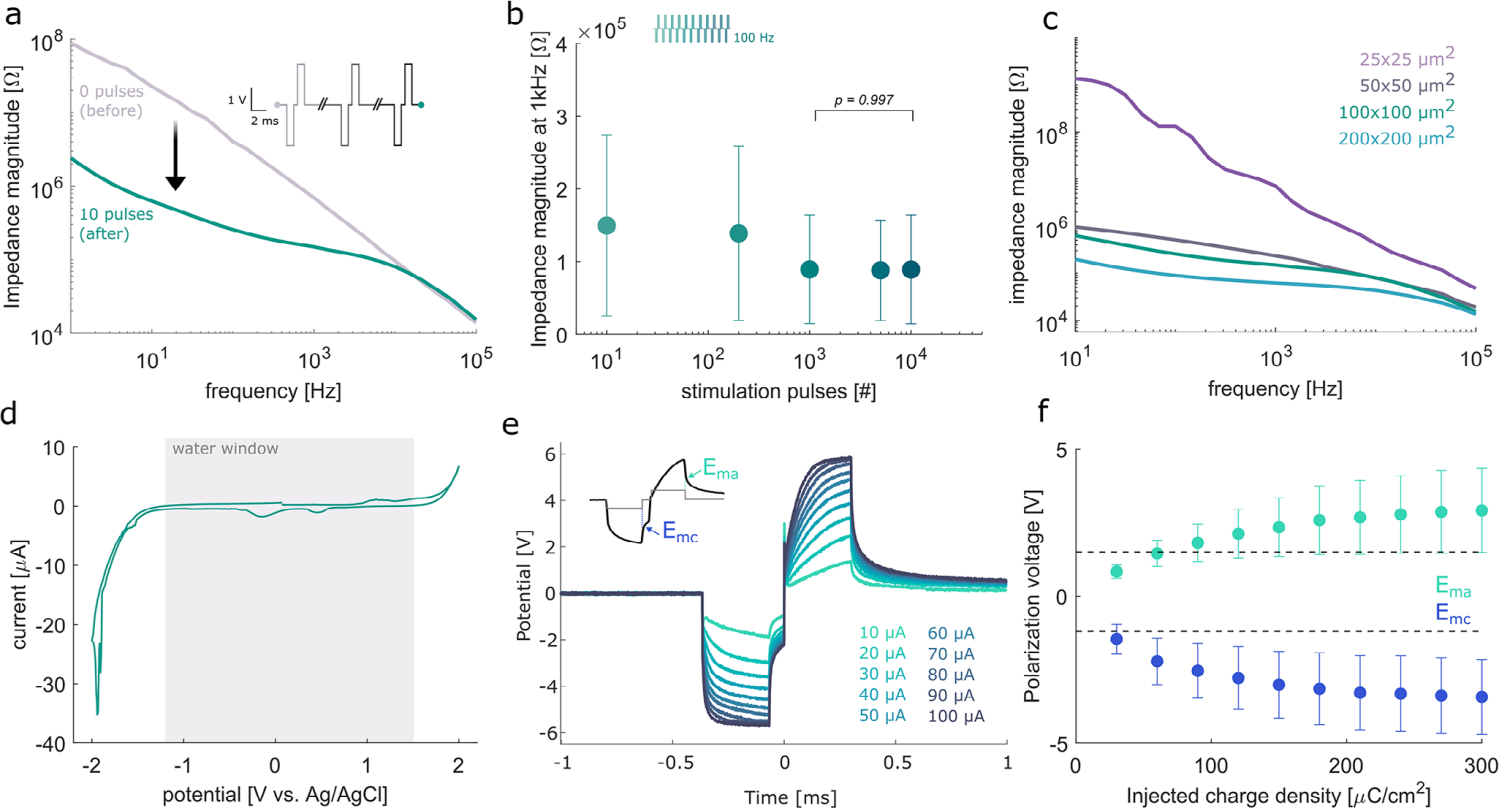
Electrochemical characterization of the electrodes (shown for 100 × 100 µm^2^ electrodes except when stated otherwise). a) Mean impedance measurement before (grey) and after (green) cleaning. b) Stimulation stability of the electrodes for up to 10^4^ pulses (mean and standard deviation). c) Mean impedance of electrodes with varying areas. All mean and standard deviations are from *n* = 6 samples. d) Exemplary cyclic voltammetry measurement indicating the water window. e) Voltage response to biphasic current pulses of amplitudes between 10–100 µA. All traces are the mean of *n* = 3 samples. The inset shows an exemplary voltage response (black) to a current pulse (grey), and the resulting determination of *E*_ma_ and *E*_mc_. f) Charge injection capacity (CIC) calculated from the voltage responses (mean and standard deviation from *n* = 3 samples).

**Figure** **3** shows the electrochemical properties for electrodes of 100 × 100 µm^2^ area. The impedance spectroscopy is shown in Figure 3a, both for pristine electrodes and after 10 voltage‐controlled pre‐conditioning biphasic pulses between +2 and −2 V. By driving the voltage beyond the water window (Figure 3d), Faradaic currents are generated at the electrodes. We can observe how the impedance magnitude decreases by several orders of magnitude after the pre‐conditioning pulses. This phenomenon has been previously observed in carbon‐based electrodes embedded in silicone or epoxy.^[^ ^84^, ^85^ ^]^ A possible explanation for this effect is that the Faradaic currents act toward removing the possible fabrication‐induced contaminations generated during laser pyrolysis, and that have adsorbed at the surface. Other studies of induced carbon electrodes have reported a voltage stimulation as an initial cleaning or stabilization step.^[^ ^73^, ^76^ ^]^ Comparing the conditioned impedance values at 1 kHz with that of planar gold electrodes on polymer substrates, we obtain a slightly lower areal impedance for the LIG electrodes of 1.49 kΩ mm^2^ compared to 1.71 kΩ mm^2^ of gold, possibly due to the rougher surface morphology.^[^ ^75^ ^]^ Carbon‐based electrodes such as chemically doped porous graphene show significantly lower specific impedances (≈0.04 kΩ mm^2^).^[^ ^75^ ^]^ One aspect that directly affects the impedance is the thickness of the porous carbon film, which is limited in this work by the thickness of the deposited parylene layer. Increasing the thickness of the parylene coating would supply more precursor material to the carbonization process, potentially reducing the resulting carbon layer's impedance. However, this would also lead to an increased device footprint, bending stiffness, and manufacturing time. Depending on the application, device designs should consider this tradeoff.

We further tested the electrochemical stability of the electrodes– when subject to 10^4^ voltage stimulation pulses outside the water window, as shown in Figure 3b. After conditioning, the mean impedance at 1 kHz was 149.6 kΩ and remained relatively stable for up to 200 pulses (decreasing by 6.4%). After 10^3^ pulses, the mean impedance decreased to 89.4 kΩ, but the decrease is found to be not significant (*p* = 0.33) due to the large variation. The high standard deviation of electrode impedance is attributed partly to the stochastic formation of 3D surface morphologies, as well as variation in debris following carbonization. After 10^4^ pulses, the impedance stabilizes, with no significant difference between 10^3^ and 10^4^ pulses (*p* = 0.997). Similar behavior to repeated stimulation pulses is reported for other pyrolyzed carbon electrodes and is often attributed to their gradual activation.^[^ ^75^, ^86^ ^]^ Figure 3c shows the influence of the nominal electrode area to the impedance, in order to assess the miniaturization limit of the chosen set of fabrication parameters. We find that the mean impedance at 1 kHz is reduced to 61.9 kΩ for electrodes of 200 × 200 µm^2^, increased to 149.6 kΩ for those of 100 × 100 µm^2^ nominal area, and to 234 kΩ for 50 × 50 µm^2^. Electrodes of 25 × 25 µm^2^ were not deemed functional, as the mean impedance at 1 kHz is higher than 1 MΩ, and the impedance spectrum is largely capacitive. This may be partially attributed to an incomplete pyrolysis due to a limited scanned area. We conclude that, currently, the smallest programmable area achievable with the proposed fabrication strategy is 50 × 50 µm^2^. One of the current limitations of laser micromachining, when compared with established techniques such as photolithography, is the feature resolution. Previously reported laser‐induced graphitic electrodes over metal structures, achieved via IR irradiation of parylene‐C over thick Pt foil, identified a downsizing limit of 200 µm diameter or larger.^[^ ^76^ ^]^ Further parameter optimization studies should be conducted with a desired final size in mind, in order to achieve smaller conductive electrode structures in the future. For all following experiments, an electrode size of 100 × 100 µm^2^ was selected as the optimal trade‐off between electrochemical impedance and adequate size for in vitro neuronal recordings.

To understand the stimulation capabilities of the electrodes, we measured the voltage response of the electrode to a balanced biphasic current pulse at increasing current amplitudes and a 300 µs phase duration (Figure 3e). From the voltage transients, it is possible to determine the electrode's charge injection capacity (CIC), which determines how much charge can be injected into the electrolyte without polarizing the electrode beyond undesired limits.^[^ ^87^ ^]^ The water window, as shown in Figure 3d, is generally considered a safe stimulation limit, as maintaining the electrode polarization within this range minimizes the formation of unwanted and irreversible reaction products, such as gaseous H_2_ and O_2_. The voltage transients can be analyzed to identify the most cathodic (*E*_mc_) and anodic (*E*_ma_) electrode polarizations, allowing comparison with the previously defined water window. To estimate *E*_mc_ and *E*_ma_, we stimulated the *n* = 3 electrodes with cathodic‐leading biphasic pulses of increasing amplitude ranging from 10 to 100 µA. We converted the stimulation current *I*_inj_ during the stimulation pulse to the injected charge per area: *Q*_inj_ = (*I*_inj_ × *t*_p_)/*A*, where *A* represents the area, and *t*_p_ the phase duration. We finally estimated the CIC as the injected charge corresponding to the intersection between the first polarization voltage *E*_ma_ or *E*_mc_, and the established water window, obtaining a CIC of 30 µC cm^−2^. This limit of safe charge density, albeit considerably lower than other carbon‐based electrodes like PEDOT: PSS (up to 2 mC cm^−2^),^[^ ^88^ ^]^ is in the order of magnitude of stimulation thresholds in many in vivo or in vitro neural stimulation applications with similarly‐sized electrodes, such as that of motor cortex,^[^ ^89^ ^]^ visual cortex,^[^ ^90^, ^91^ ^]^ or subretinal stimulation.^[^ ^92^, ^93^ ^]^

### 2.3 Imaging

**Figure 4:**
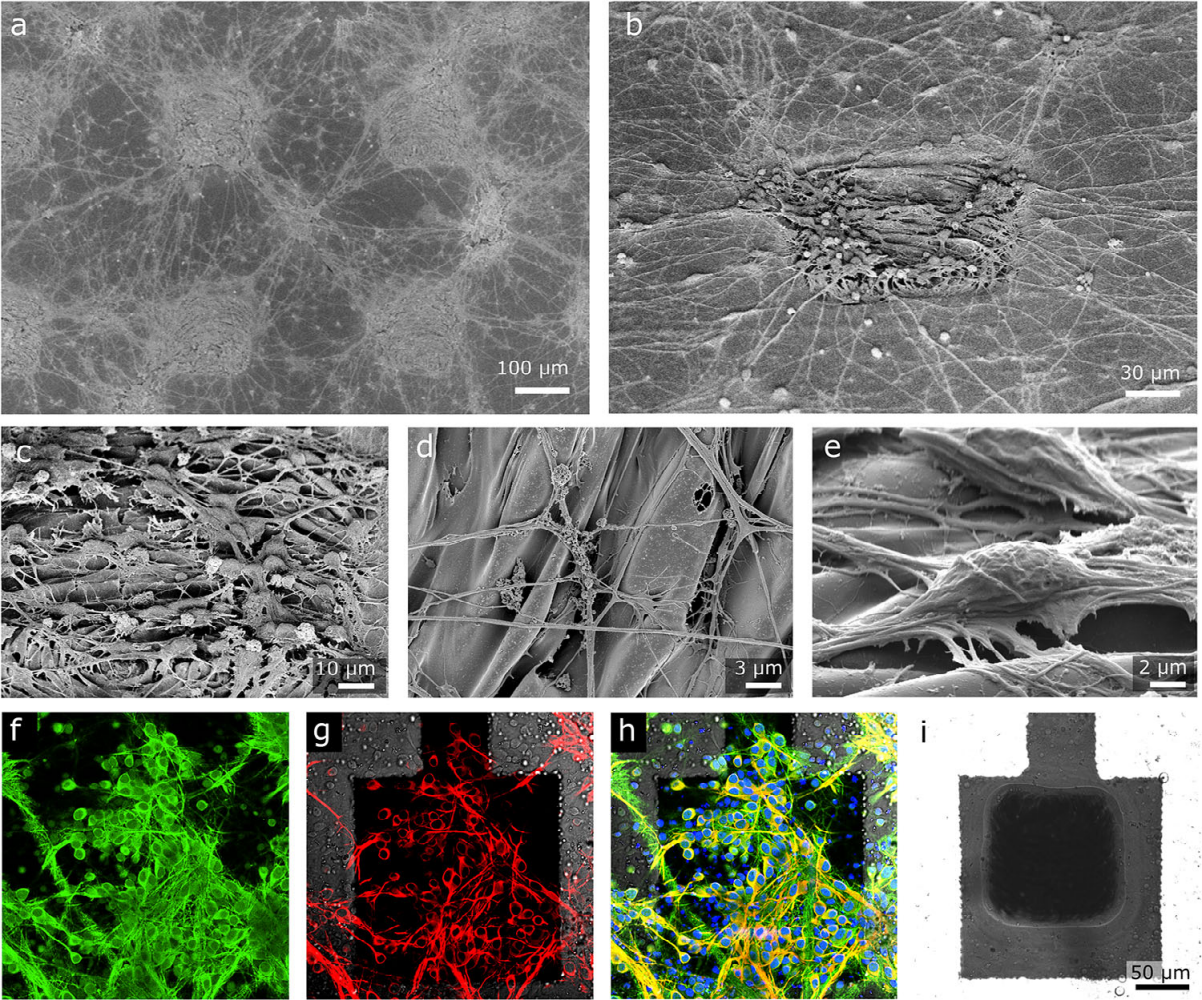
Imaging of MEA (electrode size 100 × 100 µm^2^) cultured with primary neurons. a–e) SEM images. f–i) Fluorescence microscopy images of immunochemical staining analysis: axons (f), cell bodies (g), nuclei (h, blue), underlying electrode (i).

In order to assess the suitability of the laser carbon electrodes for in vitro studies, we performed SEM of MEAs after one week of primary neuronal culture. The SEM images seen in **Figure** **4** align with other reports of the neurons’ preferential growth over rough coatings compared to the comparably flat parylene surface. Rough, 3D or nanostructured surfaces are known to positively influence cell growth and/or cell adhesion.^[^ ^94^, ^95^ ^]^ For primary neurons, it has been shown that nanostructured surfaces can enhance neurite branching.^[^ ^96^, ^97^ ^]^ Figure 4a,b shows the preferred neurite branching toward the rough carbon coating of the electrode active sites. The array of carbon electrodes can be identified on the sample surface by observing the increased aggregation of neurons and neurite branches. Close‐ups on the carbon surface (Figure 4c–e) show the rough 3D structure of the carbonaceous coating from the laser processing. We can also observe the neurite branching proliferation on the carbonaceous structures.

Next, we performed immunochemical analysis to confirm neuronal growth over the MEAs. To make a homogenous neuronal network and avoid aggregation, the primary neurons were densely seeded on the fabricated MEA. Figure 4f–h shows the staining of a primary hippocampal neuron culture at 7 days in vitro (DIV) over the MEA. MAP2‐positive cell bodies (Figure 4g, red) are present all over the surface of the MEA, irrespective of the parylene or carbon coating, with corresponding nuclei observed in blue (Figure 4h). The tau1‐positive axons (Figure 4f, green) can be observed to similarly extend over all the electrode surface, and to form a tight network of connections.

### 2.4 Neuronal Recording and Stimulation

**Figure 5:**
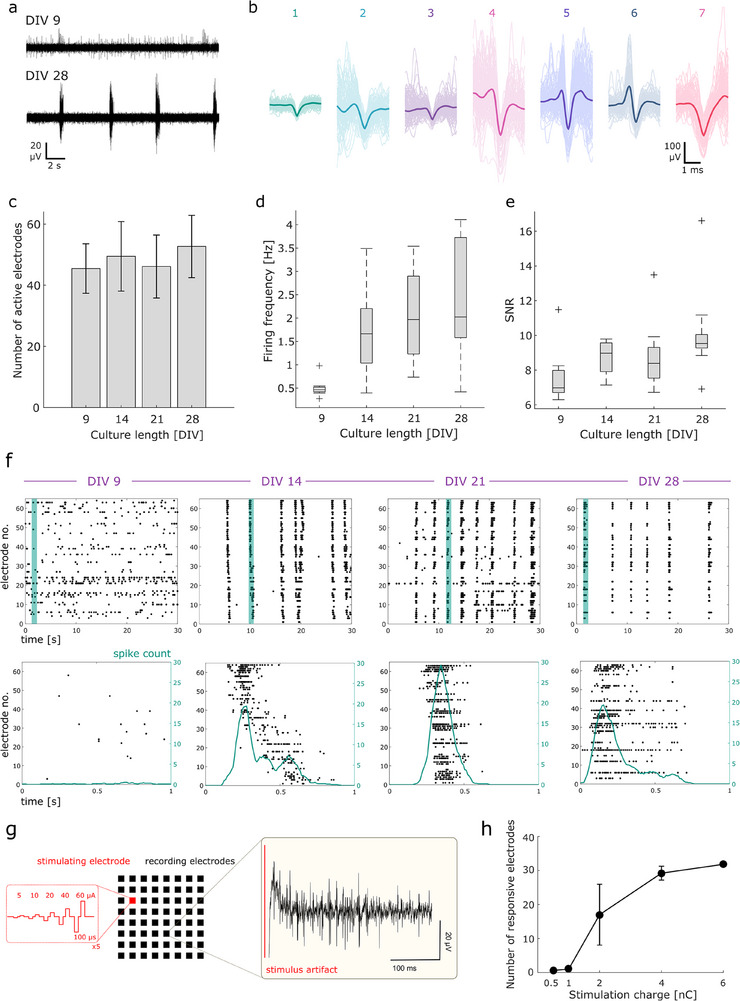
Electrophysiological MEA recording and stimulation. a) Exemplary data (bandpass‐filtered between 100–2000 Hz) at different days in culture. b) Identified clusters of spiking activity at 28 DIV. c–e) number of identified active electrodes, firing frequency, and SNR over 4 weeks, respectively (*n* = 9): c) mean and standard deviation; d,e) boxplots with outliers represented by “+”. f) Raster plots of identified spiking activity and exemplary histograms of spontaneous bursting activity. g) Stimulation protocol and exemplary data. h) Number of responsive electrodes per stimulation charge, for one MEA (*n* = 5).

We next performed electrophysiological measurements of primary neuron culture over the course of 4 weeks from 9 MEAs. The design used for the in vitro experiments is shown in Figure 1b and consists of 64 electrodes and 4 reference electrodes. Figure S5 (Supporting Information) shows phase contract images of an exemplary MEA cultured with primary hippocampal neurons at 4 DIV. Examples of spontaneous activity are shown in **Figure** **5**a. At 9 DIV, the recorded spontaneous action potentials (spikes) are sporadic, unorganized, and occurring at a low rate. At 28 DIV, the cultured neurons have established a dense neurite network, organizing the spontaneous firing into bursts. The extracellular spikes recorded from a single electrode are shown in Figure 5b. The spikes were clustered using a spike sorting algorithm.^[^ ^98^ ^]^ Deligkaris et al. suggested that the typical shape of extracellular action potentials (EAPs) recorded from the soma of a single neuron is often monophasic or biphasic, while EAPs recorded from neurites are typically triphasic, consisting of a positive, a pronounced negative peak, and a second positive peak.^[^ ^99^ ^]^ Here, given the relatively large sensing area (100 × 100 µm^2^), most of the electrodes recorded from multiple neuronal somas and neurites alike.

In order to assess the stability of the recording performance of the laser‐induced carbon MEAs, we calculated the number of active electrodes per MEA, the firing frequency of the network, and the signal‐to‐noise ratio (SNR), shown in Figure 5c–e respectively. The electrodes were considered active if they exhibited an average firing frequency higher than 0.1 Hz.^[^ ^100^ ^]^ We observed consistent performance from the MEAs over weeks, as the number of active electrodes per sample remained stable (with an overall mean of 48.4 ± 3.4 electrodes). The variation of active electrodes over time can be partly attributed to the connection between the contact pads and the pins of the recording system. Additionally, an increase in neuron‐electrode seal resistance is known to amplify extracellular spikes, thus possibly leading to variations in the active condition of single electrodes.^[^ ^101^, ^102^, ^103^ ^]^ In culture, the proliferation of neural stem cells, glial cells, and neurite branching leads to variations in seal resistance due to the high resistivity of the cell membrane.^[^ ^104^ ^]^ Electrodes with non‐planar surfaces promote such an increase in seal resistance compared to planar electrodes.^[^ ^105^, ^106^ ^]^ An improved sealing also explains the increase in SNR in Figure 5e, as a high resistance would reduce the leakage current through the gap between the cell and the substrate.^[^ ^101^, ^107^ ^]^ The average SNR measured increases from 7.58 to 10.13 for 9 DIV and 28 DIV, respectively. These values are in accordance with other reported SNR values for in vitro and in vivo recording.^[^ ^103^, ^108^, ^109^, ^110^ ^]^

The evolution of the cultured neuronal networks can be assessed by analyzing the firing frequency of the spontaneous recordings. The average firing frequency increased from 0.5 Hz to almost 2.5 Hz over the course of 4 weeks. Spontaneous bursting activity is a crucial feature of neuronal networks both in vivo and in vitro, and indicates the successful formation of excitatory and inhibitory synaptic connections in the network. Vernekar and LaPlaca (2020) monitored the bursting activity in neuronal‑astrocytic co‑cultures (2500 cells mm^−3^ plating cell density, compared to 5000 cells mm^−3^ in the present study), and found that this activity develops rapidly during the first week of culture and emerges in the second week.^[^ ^111^ ^]^ Similarly, the present study shows the development of a synchronized bursting activity within the first two weeks is slower. The MEA can steadily detect spontaneous bursts during two more weeks in culture, revealing a slight increase in firing frequency each week as found in other reports.^[^ ^112^, ^113^, ^114^ ^]^ Figure 5f shows raster plots and spiking histograms from an exemplary MEA over 4 weeks. At 9 DIV, the spontaneous EAPs are sporadic and isolated, with a maximum spike count of 0.5 spikes over a 1s interval. At 14 DIV, the spontaneous EAPs are greatly increased, and start to show an organized behavior into synchronized bursts, similar to reported.^[^ ^115^ ^]^ At this stage, the maximum spike count during a bursting event is 19.4 spikes. The increase in synchronization of spontaneous action potentials reflects the gradual organization of the neuronal network, in which neurons extend axons and dendrites and form synapses.^[^ ^116^, ^117^ ^]^ The spontaneous activity further synchronizes at 21 and 28 DIV, with a maximum spike count of 29.2 and 19.3 spikes, respectively.

We further tested a proof‐of‐principle of the stimulation capabilities of the laser‐induced carbon electrodes after 4 weeks in culture. The protocol of current‐based stimulation is shown in Figure 5g,h. A series of incremental biphasic current pulses from 5 to 60 µA distanced by 1 s was delivered to one stimulating electrode at a time, in order to assess the minimum charge threshold needed to evoke a response in the network. The inset shows the data of the evoked response from one of the recording electrodes of one MEA, with the stimulation artifact of one electrode indicated in red. The same spike detection algorithm as before was applied to identify action potentials within a window of 300 ms after stimulus onset excluding the stimulus artifact. The resulting activation curve of an exemplary MEA is shown in Figure 5h. At the charge of 0.5 and 1 nC, almost no response is evoked. At 2 nC delivered charge or higher, a non‐negligible number of active recording electrodes were classified as responsive (more than 2 spikes detected). The high variance at 2 nC suggests that the minimum stimulation charge threshold for the network is ≈2 nC, with some electrodes already recording evoked activity while others receive no evoked response. This charge corresponds to 20 µC cm^−2^, within the safe charge injection limits identified in Figure 3f. Above such threshold, the number of active electrodes that recorded evoked activity increases visibly, as seen also from the raster plots in Figure S4 (Supporting Information). Results from the other 3 tested MEAs reveal variability in the number of responsive electrodes recruited, but a similar threshold behavior ≈2 nC (Figure S4, Supporting Information).

## 3 Conclusion

We have shown the successful fabrication of an array of carbon‐based electrodes fabricated by laser‐induced carbonization of parylene directly over thin metal layers. The proposed fabrication allows for one‐step electrode opening of the desired area by converting the insulating polymer into a conductive carbon‐based coating electrically connected to the metal circuitry. The MEA device is entirely patterned using a UV laser (355 nm), used both for the metal ablation and for the laser carbonization steps. We have investigated the optimal laser parameters to obtain the smallest carbon electrode area, observing that a slow scan speed and a dense line pitch are beneficial for the conversion process. Electrochemical measurements confirmed the stability of the sensors under 10 000 voltage‐controlled stimulation pulses. Immunostaining and SEM imaging with primary neuronal cultures confirmed the suitability of the proposed sensors for in vitro studies and showed good coverage of the neurons over the rough carbon. Last, electrophysiological MEA recordings of primary neuronal cultures showed the stability of the recording performance over at least 4 weeks in culture, together with a proof‐of‐principle of the stimulation capabilities. We propose the presented one‐step laser conversion of a parylene‐C coating into a conductive carbon‐based coating as a rapid, precise, and cost‐effective fabrication protocol, suitable for neuroelectronics and bioelectronics devices. These results suggest that the presented fabrication method can be further tested in thin‐film electrode designs for in vivo applications. Additionally, other polymer precursors, such as polyimide, can be investigated in future studies for similar applications.

## 4 Experimental Section

### Materials

2‐Propanol (≥99.5%) and ethanol (≥99.5%) were obtained from Carl Roth (Germany). Deionized water was generated by a water purification system (Berry Tech, Germany). Electrochemical measurements were performed in phosphate‐buffered saline (PBS) purchased from Sigma‐Aldrich (St. Louis, USA). Parylene‐C dimer (dichlorodi‐p‐xylylene) was obtained from Specialty Coating Systems (USA). For immunochemical analysis, Anti‐Tau1 antibody was purchased from Novus Biologicals (USA), Anti‐MAP2 from Merck Millipore (USA), the secondary antibodies and Hoechst33342 from Thermo Fisher Scientific (USA), TritonX and BSA from Sigma‐Aldrich (USA).

### Device Fabrication

Glass substrates (49 × 49 mm) were cleaned with acetone and isopropanol. Parylene‐C (5 µm) was deposited via chemical vapor deposition (PDS 2010, SCS LabcoaterTM 2 Parylene Deposition System, Specialty Coating Systems, USA) over the glass. 15 nm of Ti and 100 nm of Au were sputtered (nanoPDV, Moorefield, UK), and then patterned via laser ablation using a UV nanosecond pulsed laser scanner (MD‐U1000, Keyence, Osaka, Japan) with the following parameters: 5% power, 500 mm s^−1^ scan speed, 60 kHz shutter frequency, 0 mm defocus, 1 repetition, 10 µm line pitch (corresponding to 1.3 µJ per pulse). The contact pads were masked with Kapton tape or polydimethylsiloxane (PDMS), and a second layer of 5 µm parylene‐C was deposited. The electrode areas were then opened via laser pyrolysis of parylene‐C, performed at 1 mm s^−1^ scan speed, 400 kHz shutter frequency, 0.2% power, 1 repetition, 2 µm line pitch, and 4 mm defocus (corresponding to 0.22 µJ per pulse), and the contact masks were peeled off gently. For the free‐standing electrodes, the outline of the electrodes was laser‐cut with a 500 mm s^−1^ scan speed, 40 kHz shutter frequency, 15% power, 40 repetition, and 0 mm defocus with the same laser system (10 µJ per pulse). All samples were gently washed with DI water and blow‐dried afterward.

### Electrochemical Characterization

All electrochemical measurements were performed using a VSP‐300 potentiostat (BioLogic Instruments, France) in a 3‐electrode setup, with an Ag/AgCl (3 м NaCl) reference electrode, and a coiled platinum wire as the counter electrode. Open circuit potential (OCP), electrochemical impedance spectroscopy (EIS), cyclic voltammetry (CV), and chronoamperometry (CA) were used to determine the electrochemical performance of the sensors. First, the OCP was measured versus Ag/AgCl for 60 s. EIS was then measured from 1–10^5^ Hz with a signal of 10 mV amplitude. CV was run between ±1 V versus Ag/AgCl at 200 mV s^−1^ for 3 cycles. The water window was measured with a wide CV (±2 V vs Ag/AgCl, 1 V s^−1^) for 3 cycles. Then, a cleaning/wetting step was performed by applying 10 biphasic stimulation pulses of ±2 V, 1 ms, 100 Hz through CA. The OCP was measured again for 60 s, as well as the EIS (1–10^5^ Hz) (10 mV or 20 mV amplitude) and CV (±1 V vs Ag/AgCl at 200 mV s^−1^, 3 cycles). To test the electrochemical stability of the electrodes during stimulation pulses, 10^4^ biphasic pulses of ±2 V with 1 ms/phase at 100 Hz were applied in a 3‐electrode setup. All data was processed in MATLAB (MATLAB R2023a, MathWorks, USA). The voltage response of the electrodes to biphasic current pulses was recorded with an oscilloscope (InfiniiVision DSOX2024A, Keysight, USA). The current pulses were applied with an electrophysiology stimulator/amplifier chip (RHS2116, Intan Technologies, USA) in the three‐electrode setup described above. The phase of each pulse had a duration of 300 µs (leading cathodic) with a 50 µs interphase delay. The amplitude of the pulses ranged between 10 and 100 µA in steps of 10 µA.

### Device Imaging

The optical images and the 3D scans were obtained with a 3D laser scanning microscope (20X, VK‐X250, Keyence) and the profiles analyzed in MATLAB.

### Cell Culture

Primary hippocampal neurons were dissected from the hippocampi of 18‐day‐old Wistar rat embryos (Charles River Laboratories). The tissues were dissociated into single cells using trypsin. The dissociated cells were suspended at a cell density of 5 × 10^6^ cells mL^−1^ in a culture medium. The culture medium for neurons was Neurobasal medium (Thermo Fisher Scientific) supplemented with 0.5 × 10^−3^ м glutamine (Sigma‐Aldrich), 25 × 10^−6^ м glutamine (Sigma‐Aldrich), 50 µg mL^−1^ gentamicin (Thermo Fisher Scientific), and 2% B‐27 supplement (Thermo Fisher Scientific). The MEAs were sterilized in UV light for 2 min, and then coated with polyethyleneimine (PEI) overnight. After removal and washing of the PEI, the surface of the MEA was treated with a solution of laminin for 30 min, excluding the reference electrodes. After removal of laminin, the cells were seeded and the samples stored in an incubator for 30 min before adding culture medium. All animal experiments were approved by the Biological Safety and Ethics Committee of NTT Basic Research Laboratories (approval ID 2023‐02), which follow the Guidelines for the Proper Conduct of Animal Experiments of the Science Council of Japan (Kohyo‐20‐k16‐2, 2006).

### Immunostaining

To fixate the samples for immunostaining, the culture medium was replaced with 4% paraformaldehyde (PFA) diluted in PBS, for 30 min at room temperature. The solution was gently removed, exchanged with 1% Bovine serum albumin (BSA) blocking buffer and 0.1% TritonX surfactant, and left at 4 °C for 3 h. The solution was then exchanged with a 0.1% BSA solution containing 1:250 to 1:500 primary antibodies. The samples were left to incubate overnight at 4 °C, then washed with PBS. For secondary antibody incubation, a solution of 0.1% BSA and 0.7% antibody was used and the samples were left at 4 °C for 6 h and then washed in PBS. The images were obtained using a confocal fluorescence microscope (SD‐OSR, Olympus, Japan).

### SEM/FIB Imaging

To fixate the samples for SEM imaging, the culture medium was replaced with 2% paraformaldehyde (PFA) diluted in PBS for 30 min. Then, the solution was gradually replaced with ethanol by repeatedly exchanging it with a mixture of ethanol and water. Afterward, the solution was exchanged with tert‐butyl alcohol and freeze‐dried overnight. After the freeze‐drying, 10‐nm thickness of platinum was sputtered on the sample. The sample was then observed using the focused ion beam (FIB)‐SEM system (Auriga 60 Cross Beam Workstation, Carl Zeiss, Germany). All SEM observation was performed at an acceleration voltage of 5 kV.

### Electrophysiological Experiments

Electrical recording and stimulation were performed using an MEA control system (MED64‐Basic System, Alpha Med Scientific, Japan), which consisted of a main amplifier, a head amplifier, and a connector. The MEAs were designed with an electrode area of 100 × 100 µm^2^, and were fabricated with the selected laser parameters of 400 kHz frequency, 0.2% power, 4 mm defocus, 1 repetition, 1 mm s^−1^, and 0.002 mm line pitch. Each MEA was connected to the amplifiers via the connector. The recorded signals were amplified with a gain of 2000 and passed through a band‐pass filter at 100–2000 Hz. The analog signals were digitized at 20 kHz. The recording environment was maintained at 37 °C and 5% CO_2_ using a stage‐top mini‐incubator (TOKAI HIT, Japan). The sample with the MEA was immediately moved from the culture incubator to the recording mini‐incubator. Additionally, it was incubated for 5 min before recording experiments to stabilize the recording environment and reduce the variance of neuronal activities. Spontaneous activities were recorded for 10 min every 7 days from 9 DIV. Spontaneous activities of neurons were analyzed using a custom MATLAB code. A 10 min trace containing spontaneous activity was used to analyze characteristics, including the number of active electrodes, firing frequency, and SNR. The characteristics were calculated from the traces of 64 electrodes and then averaged for each MEA. The representative value at each DIV was obtained by averaging the value of multiple MEAs. The process of analyzing spontaneous activities was as follows. Negative peaks beyond a threshold (5× standard deviation) were detected as spike activities. For spike sorting, the spikes were sorted into spike trains of individual neurons using a spike sorting method as previously reported.^[^ ^98^, ^118^ ^]^ Active electrodes were considered as those presenting a firing frequency (spikes per second) higher than 0.1 Hz. For SNR calculation, the mean spike amplitude from each electrode was divided by the standard deviation of the noise.^[^ ^108^ ^]^

Electrical stimulation experiments were performed on 4 MEAs at 35 DIV. From one stimulating electrode at a time, a series of biphasic pulses of varying current amplitude (5–60 µA) were delivered with a phase duration of 100 µs, an inter‐pulse interval of 1 s, and repeated 5 times after a 5 s interval. All recorded data was processed in MATLAB. The stimulus artifact was excluded from the data using the known timestamps and an artifact window of 30 ms. Active electrodes were considered as those presenting a firing frequency (spikes per second) higher than 0.1 Hz, and responsive electrodes were considered as those of the active ones presenting at least 2 spikes detected within a window of 300 ms after the artifact. The histogram data were obtained with bins of 10 ms, and the spike counts were averaged among the 5 trial repetitions among all active recording electrodes.

### Statistical Analysis

All data were expressed as mean ± standard deviation (SD). All statistical analyses were performed using MATLAB Statistics and Machine Learning Toolbox. The significance analysis was performed with a two‐sample *t*‐test using the in‐built MATLAB function “ttest2”. The interquartile range and outliers were computed using the MATLAB function “boxplot”. Specifically, the outliers were calculated as any data point being greater than *q*_3_ + 1.5 × (*q*_3_ −* q*_1_) or less than *q*_1_ − 1.5 × (*q*_3_ − *q*_1_), with *q*_1_ and *q*_3_ being the 25th and 75th percentiles of the sample data, respectively.

## Conflict of Interest

The authors declare no conflict of interest.

## Author Contributions

Conceptualization was done by F.D.D., K.S., and B.W. Methodology was done by F.D.D., K.S., and B.W. Establishment of experimental techniques was done by F.D.D., K.S., B.D.C., T.G., D.T., L.H., G.A.B., H.P., and T.T. Original draft was written by F.D.D., K.S., and B.W. Writing the review and editing were done by all authors. All authors have read and agreed to the published version of the manuscript.

## Supporting information



Supporting Information

## Data Availability

The data that support the findings of this study are available from the corresponding author upon reasonable request.;

## References

[1] X. Lyu , Y. Hu , S. Shi , S. Wang , H. Li , Y. Wang , K. Zhou , Biosensors 2023, 13, 815. 37622901 10.3390/bios13080815 PMC10452556

[2] Y. Wang , H. Haick , S. Guo , C. Wang , S. Lee , T. Yokota , T. Someya , Chem. Soc. Rev. 2022, 51, 3759. 35420617 10.1039/d2cs00207h

[3] H. Mirzajani , M. Kraft , ACS Sens. 2024, 9, 4328. 39239948 10.1021/acssensors.4c00442

[4] P. Li , G.‐H. Lee , S. Y. Kim , S. Y. Kwon , H.‐R. Kim , S. Park , ACS Nano 2021, 15, 1960. 33534541 10.1021/acsnano.0c06688

[5] S.‐H. Sunwoo , S. I. Han , C. S. Park , J. H. Kim , J. S. Georgiou , S.‐P. Lee , D.‐H. Kim , T. Hyeon , Nat. Rev. Bioeng. 2024, 2, 8.

[6] S. Oh , J. Jekal , J. Liu , J. Kim , J.‐U. Park , T. Lee , K.‐I. Jang , Adv. Funct. Mater. 2024, 34, 2403562.

[7] A. Jonsson , Z. Song , D. Nilsson , B. A. Meyerson , D. T. Simon , B. Linderoth , M. Berggren , Sci. Adv. 2015, 1, 1500039. 10.1126/sciadv.1500039 PMC4640645 26601181

[8] G. Schiavone , F. Fallegger , X. Kang , B. Barra , N. Vachicouras , E. Roussinova , I. Furfaro , S. Jiguet , I. Seáñez , S. Borgognon , A. Rowald , Q. Li , C. Qin , E. Bézard , J. Bloch , G. Courtine , M. Capogrosso , S. P. Lacour , Adv. Mater. 2020, 32, 1906512. 10.1002/adma.201906512 32173913

[9] M. Levin , J. Selberg , M. Rolandi , iScience 2019, 22, 519. 31837520 10.1016/j.isci.2019.11.023 PMC6920204

[10] H. Li , N. Asefifeyzabadi , K. Schorger , P. Baniya , H. Yang , M. Tebyani , A. Barbee , W. S. Hee , A. Gallegos , K. Zhu , C. Recendez , F. Lu , G. Luka , S. Kim , K. Devarajan , T. Nguyen , S. Figuerres , C. Franco , E. Aslankoohi , H. Carrión , N. Norouzi , M. Gomez , M. Zhao , R. R. Isseroff , M. Teodorescu , M. Rolandi , Adv. Mater. Technol. 2025, 70039.

[11] J. Criscione , Z. Rezaei , C. M. Hernandez Cantu , S. Murphy , S. R. Shin , D.‐H. Kim , Biosens. Bioelectron. 2023, 220, 114840. 36402101 10.1016/j.bios.2022.114840

[12] J. U. Lind , M. Yadid , I. Perkins , B. B. O'Connor , F. Eweje , C. O. Chantre , M. A. Hemphill , H. Yuan , P. H. Campbell , J. J. Vlassak , K. K. Parker , Lab Chip 2017, 17, 3692. 28976521 10.1039/c7lc00740j PMC5810940

[13] M. Eichler , H.‐G. Jahnke , D. Krinke , A. Müller , S. Schmidt , R. Azendorf , A. A. Robitzki , Biosens. Bioelectron. 2015, 67, 582. 25445619 10.1016/j.bios.2014.09.049

[14] P. Cavassin , A.‐M. Pappa , C. Pitsalidis , H. F. P. Barbosa , R. Colucci , J. Saez , Y. Tuchman , A. Salleo , G. C. Faria , R. M. Owens , Adv. Mater. Technol. 2020, 5, 1900680.

[15] J. Kim , I. Jeerapan , J. R. Sempionatto , A. Barfidokht , R. K. Mishra , A. S. Campbell , L. J. Hubble , J. Wang , Acc. Chem. Res. 2018, 51, 2820. 30398344 10.1021/acs.accounts.8b00451 PMC8183421

[16] C. Choi , Y. Lee , K. W. Cho , J. H. Koo , D.‐H. Kim , Acc. Chem. Res. 2019, 52, 73. 30586292 10.1021/acs.accounts.8b00491

[17] C. Wang , E. Shirzaei Sani , W. Gao , Adv. Funct. Mater. 2022, 32, 2111022. 36186921 10.1002/adfm.202111022 PMC9518812

[18] C. Lim , Y. J. Hong , J. Jung , Y. Shin , S.‐H. Sunwoo , S. Baik , O. K. Park , S. H. Choi , T. Hyeon , J. H. Kim , S. Lee , D.‐H. Kim , Sci. Adv. 2021, 7, abd3716. 10.1126/sciadv.abd3716 PMC8104866 33962955

[19] R. K. Mishra , J. R. Sempionatto , Z. Li , C. Brown , N. M. Galdino , R. Shah , S. Liu , L. J. Hubble , K. Bagot , S. Tapert , J. Wang , Talanta 2020, 211, 120757. 32070607 10.1016/j.talanta.2020.120757

[20] T. Arakawa , K. Tomoto , H. Nitta , K. Toma , S. Takeuchi , T. Sekita , S. Minakuchi , K. Mitsubayashi , Anal. Chem. 2020, 92, 12201. 32927955 10.1021/acs.analchem.0c01201

[21] P. R. Patel , E. J. Welle , J. G. Letner , H. Shen , A. J. Bullard , C. M. Caldwell , A. Vega‐Medina , J. M. Richie , H. E. Thayer , P. G. Patil , D. Cai , C. A. Chestek , J. Neural Eng. 2023, 20, 014001. 10.1088/1741-2552/acab86 PMC9954796 36595323

[22] A. C. Paulk , Y. Kfir , A. R. Khanna , M. L. Mustroph , E. M. Trautmann , D. J. Soper , S. D. Stavisky , M. Welkenhuysen , B. Dutta , K. V. Shenoy , L. R. Hochberg , R. M. Richardson , Z. M. Williams , S. S. Cash , Nat. Neurosci. 2022, 25, 252. 35102333 10.1038/s41593-021-00997-0

[23] H. Shin , Y. Son , U. Chae , J. Kim , N. Choi , H. J. Lee , J. Woo , Y. Cho , S. H. Yang , C. J. Lee , I.‐J. Cho , Nat. Commun. 2019, 10, 3777. 31439845 10.1038/s41467-019-11628-5 PMC6706395

[24] H. Shin , S. Jeong , J.‐H. Lee , W. Sun , N. Choi , I.‐J. Cho , Nat. Commun. 2021, 12, 492. 33479237 10.1038/s41467-020-20763-3 PMC7820464

[25] Nat. Mater. 2013, 12, 591.23782982

[26] E. Song , J. Li , J. A. Rogers , APL Mater. 2019, 7, 050902.

[27] F. Fallegger , G. Schiavone , S. P. Lacour , Adv. Mater. 2020, 32, 1903904. 10.1002/adma.201903904 31608508

[28] E. Song , J. Li , S. M. Won , W. Bai , J. A. Rogers , Nat. Mater. 2020, 19, 590. 32461684 10.1038/s41563-020-0679-7

[29] N. Obidin , F. Tasnim , C. Dagdeviren , Adv. Mater. 2020, 32, 1901482. 10.1002/adma.201901482 31206827

[30] M. Cho , J.‐K. Han , J. Suh , J. J. Kim , J. R. Ryu , I. S. Min , M. Sang , S. Lim , T. S. Kim , K. Kim , K. Kang , K. Hwang , K. Kim , E.‐B. Hong , M.‐H. Nam , J. Kim , Y. M. Song , G. J. Lee , I.‐J. Cho , K. J. Yu , Nat. Commun. 2024, 1, 2000. 10.1038/s41467-024-45803-0 PMC10917781 38448437

[31] J. J. Park , M. Kim , S. H. Ko , In Mechanics of Flexible and Stretchable Electronics, John Wiley and Sons, Ltd, Hoboken, NJ 2024, p. 207.

[32] P. Oldroyd , G. G. Malliaras , Acta Biomater. 2022, 139, 65. 34020055 10.1016/j.actbio.2021.05.004

[33] J. C. Barrese , J. Aceros , J. P. Donoghue , J. Neural Eng. 2016, 13, 026003. 26824680 10.1088/1741-2560/13/2/026003 PMC4854331

[34] R. Caldwell , M. G. Street , R. Sharma , P. Takmakov , B. Baker , L. Rieth , Biomaterials 2020, 232, 119731. 31918225 10.1016/j.biomaterials.2019.119731

[35] P. Čvančara , T. Boretius , V. M. López‐Álvarez , P. Maciejasz , D. Andreu , S. Raspopovic , F. Petrini , S. Micera , G. Granata , E. Fernandez , P. M. Rossini , K. Yoshida , W. Jensen , J.‐L. Divoux , D. Guiraud , X. Navarro , T. Stieglitz , J. Neural Eng. 2020, 17, 046006. 32512544 10.1088/1741-2552/ab9a9a

[36] J. A. Goding , A. D. Gilmour , U. A. Aregueta‐Robles , E. A. Hasan , R. A. Green , Adv. Funct. Mater. 2018, 28, 1702969.

[37] P. Oldroyd , J. Gurke , G. G. Malliaras , Adv. Funct. Mater. 2023, 33, 2208881.

[38] J. Li , M. Li , L. Tian , Y. Qiu , Q. Yu , X. Wang , R. Guo , Q. He , Int. J. Pharm. 2020, 578, 119122. 32035259 10.1016/j.ijpharm.2020.119122

[39] M. Devi , M. Vomero , E. Fuhrer , E. Castagnola , C. Gueli , S. Nimbalkar , M. Hirabayashi , S. Kassegne , T. Stieglitz , S. Sharma , J. Neural Eng. 2021, 18, 041007. 10.1088/1741-2552/ac1e45 34404037

[40] S. Sim , H. Shin , K. Bae , H. Han , Y. Kang , J. Woo , Y. Cho , I.‐J. Cho , J. Kim , Sens. Actuators, B 2023, 393, 134124.

[41] A. Wang , D. Jung , D. Lee , H. Wang , ACS Appl. Electron. Mater. 2021, 3, 5226.

[42] R. Liu , Z.‐Y. Feng , D. Li , B. Jin , Y. Lan , L.‐Y. Meng , TrAC, Trends Anal. Chem. 2022, 148, 116541.

[43] A. Asif , A. Heiskanen , J. Emnéus , S. S. Keller , Electrochim. Acta 2021, 379, 138122.

[44] S. Vasudevan , J. Kajtez , A. Heiskanen , J. Emnéus , S. S. Keller , In 2020 IEEE 33rd Int. Conf. Micro Electro Mechanical Systems (MEMS) , IEEE, NewYork City, NY, USA, 2020, pp. 388.

[45] N. Støvring , A. R. Heiskanen , J. Emnéus , S. S. Keller , ACS Appl. Mater. Interfaces 2025, 17, 14375. 39969911 10.1021/acsami.4c18998 PMC11892468

[46] S. Ranganathan , R. McCreery , S. M. Majji , M. Madou , J. Electrochem. Soc. 2000, 147, 277.

[47] L. Amato , A. Heiskanen , C. Caviglia , F. Shah , K. Zór , M. Skolimowski , M. Madou , L. Gammelgaard , R. Hansen , E. G. Seiz , M. Ramos , T. R. Moreno , A. Martínez‐Serrano , S. S. Keller , J. Emnéus , Adv. Funct. Mater. 2014, 24, 7042.

[48] P. Kusumanchi , J. G. Madsen , T. Bek , S. S. Keller , R. S. Davidsen , Biomed. Microdevices 2025, 27, 7. 39934449 10.1007/s10544-024-00729-8 PMC11813987

[49] N. Støvring , B. Rezaei , A. Heiskanen , J. Emnéus , S. S. Keller , Micro Nano Eng. 2024, 23, 100257.

[50] M. Devi , H. Wang , S. Moon , S. Sharma , V. Strauss , Adv. Mater. 2023, 35, 2211054. 10.1002/adma.202211054 36841955

[51] J. Lin , Z. Peng , Y. Liu , F. Ruiz‐Zepeda , R. Ye , E. L. G. Samuel , M. J. Yacaman , B. I. Yakobson , J. M. Tour , Nat. Commun. 2014, 5, 5714. 25493446 10.1038/ncomms6714 PMC4264682

[52] R. Ye , D. K. James , J. M. Tour , Adv. Mater. 2019, 31, 1803621. 10.1002/adma.201803621 30368919

[53] T.‐S. D. Le , S. Park , J. An , P. S. Lee , Y.‐J. Kim , Adv. Funct. Mater. 2019, 29, 1902771.

[54] M. Devi , S. Rawat , S. Sharma , Oxford Open Mater. Sci. 2020, 1, itab014.

[55] L. Lei , Z. Cao , J. Li , H. Hu , D. Ho , ACS Appl. Energy Mater. 2022, 5, 12790.

[56] L. Zhao , Z. Liu , D. Chen , F. Liu , Z. Yang , X. Li , H. Yu , H. Liu , W. Zhou , Nano‐Micro Lett. 2021, 13, 49. 10.1007/s40820-020-00577-0 PMC8187667 34138243

[57] A. Svetlova , H. T. J. Law , D. Kim , N. Enriquez , A. Soleimani , A. Al‐Shami , S. Kohan , R. Peck , O. E. Eremina , C. Zavaleta , M. P. S. Mousavi , M. L. McCain , Adv. Funct. Mater. 2025, 35, 2417184. 10.1002/adfm.202417184 PMC13271665 42312246

[58] S. Moon , E. Senokos , V. Trouillet , F. F. Loeffler , V. Strauss , Nanoscale 2024, 16, 8627. 38606506 10.1039/d4nr00588k PMC11064777

[59] H. Wang , P. Jiménez‐Calvo , M. Hepp , M. A. Isaacs , C. Otieno Ogolla , I. Below‐Lutz , B. Butz , V. Strauss , ACS Appl. Nano Mater. 2023, 6, 966.

[60] M. Hepp , H. Wang , K. Derr , S. Delacroix , S. Ronneberger , F. F. Loeffler , B. Butz , V. Strauss , npj Flexible Electron. 2022, 6, 3.

[61] M. d'Amora , A. Lamberti , M. Fontana , S. Giordani , J. Phys. Mater. 2020, 3, 034008.

[62] W. Wang , B. Han , Y. Zhang , Q. Li , Y.‐L. Zhang , D.‐D. Han , H.‐B. Sun , Adv. Funct. Mater. 2021, 31, 2006179.

[63] X. Huang , H. Li , J. Li , L. Huang , K. Yao , C. K. Yiu , Y. Liu , T. H. Wong , D. Li , M. Wu , Y. Huang , Z. Gao , J. Zhou , Y. Gao , J. Li , Y. Jiao , R. Shi , B. Zhang , B. Hu , Q. Guo , E. Song , R. Ye , X. Yu , Nano Lett. 2022, 22, 3447. 35411774 10.1021/acs.nanolett.2c00864

[64] M. Khatib , E. T. Zhao , S. Wei , A. Abramson , E. S. Bishop , C.‐H. Chen , A.‐L. Thomas , C. Xu , J. Park , Y. Lee , R. Hamnett , W. Yu , S. E. Root , L. Yuan , D. Chakhtoura , K. K. Kim , D. Zhong , Y. Nishio , C. Zhao , C. Wu , Y. Jiang , A. Zhang , J. Li , W. Wang , F. Salimi‐Jazi , T. A. Rafeeqi , N. M. Hemed , J. B.‐H. Tok , X. Chen , J. A. Kaltschmidt , et al., bioRxiv 2023.

[65] H. Wang , Z. Zhao , P. Liu , X. Guo , npj Flexible Electron. 2022, 6, 26.

[66] C. H. Dreimol , H. Guo , M. Ritter , T. Keplinger , Y. Ding , R. Günther , E. Poloni , I. Burgert , G. Panzarasa , Nat. Commun. 2022, 13, 3680. 35760793 10.1038/s41467-022-31283-7 PMC9237073

[67] H. Moon , B. Ryu , Int. J. of Precis. Eng. Manuf.‐Green Tech. 2024, 11, 1279.

[68] Y. Chyan , R. Ye , Y. Li , S. P. Singh , C. J. Arnusch , J. M. Tour , ACS Nano 2018, 12, 2176. 29436816 10.1021/acsnano.7b08539

[69] R. Correia , J. Deuermeier , M. R. Correia , J. Vaz Pinto , J. Coelho , E. Fortunato , R. Martins , ACS Appl. Mater. Interfaces 2022, 14, 46427. 36209418 10.1021/acsami.2c09667 PMC9585513

[70] G. W. Gross , IEEE Trans. Biomed. Eng. 1979, BME‐26, 273. 10.1109/tbme.1979.326402 447356

[71] G. W. Gross , E. Rieske , G. W. Kreutzberg , A. Meyer , Neurosci. Lett. 1977, 6, 101. 19605037 10.1016/0304-3940(77)90003-9

[72] A. F. Carvalho , A. J. S. Fernandes , C. Leitão , J. Deuermeier , A. C. Marques , R. Martins , E. Fortunato , F. M. Costa , Adv. Funct. Mater. 2018, 28, 1805271.

[73] N. F. Santos , S. O. Pereira , A. Moreira , A. V. Girão , A. F. Carvalho , A. J. S. Fernandes , F. M. Costa , Adv. Mater. Technol. 2021, 6, 2100007.

[74] J. B. Fortin , T.‐M. Lu , Thin Solid Films 2001, 397, 223.

[75] Y. Lu , H. Lyu , A. G. Richardson , T. H. Lucas , D. Kuzum , Sci. Rep. 2016, 6, 33526. 27642117 10.1038/srep33526 PMC5027596

[76] M. Vomero , A. Oliveira , D. Ashouri , M. Eickenscheidt , T. Stieglitz , Sci. Rep. 2018, 8, 14749. 30283015 10.1038/s41598-018-33083-w PMC6170440

[77] A. Oliveira , J. S. Ordonez , D. A. Vajari , M. Eickenscheidt , T. Stieglitz , Eur. J. Transl. Myol. 2016, 26, 6062. 27990233 10.4081/ejtm.2016.6062 PMC5128966

[78] F. Zurita , S. Freko , L. Hiendlmeier , F. Del Duca , T. Groll , O. Seelbach , K. Steiger , B. Wolfrum , Adv. NanoBiomed. Res. 2024, 4, 2300102.

[79] M. Ozdemir , H. Sadikoglu , Trends Food Sci. Technol. 1998, 9, 159.

[80] M. A. Fadel , N. A. Kamel , M. M. Darwish , S. L. A. El‐Messieh , K. N. Abd‐El‐Nour , W. A. Khalil , Prog. Biomater. 2020, 9, 107. 32627137 10.1007/s40204-020-00134-3 PMC7544811

[81] T. Lippert , In Laser‐Surface Interactions for New Materials Production: Tailoring Structure and Properties, (Eds.: Miotello, A. ; Ossi, P. M. ), Springer, Berlin, Heidelberg, 2010, pp. 141–175.

[82] B. Cardenas‐Benitez , C. Eschenbaum , D. Mager , J. G. Korvink , M. J. Madou , U. Lemmer , I. D. Leon , S. O. Martinez‐Chapa , Microsyst. Nanoeng. 2019, 5, 38. 31636928 10.1038/s41378-019-0079-9 PMC6799819

[83] R. Natu , M. Islam , J. Gilmore , R. Martinez‐Duarte , J. Anal. Appl. Pyrolysis 2018, 131, 17.

[84] S.‐R. Yeh , Y.‐C. Chen , H.‐C. Su , T.‐R. Yew , H.‐H. Kao , Y.‐T. Lee , T.‐A. Liu , H. Chen , Y.‐C. Chang , P. Chang , H. Chen , Langmuir 2009, 25, 7718. 19563234 10.1021/la900264x

[85] K. Terkan , F. Zurita , T. Jamal Khalaf , P. Rinklin , T. Teshima , T. Kohl , B. Wolfrum , APL Mater. 2020, 8, 101111.

[86] M. Vomero , E. Castagnola , J. S. Ordonez , S. Carli , E. Zucchini , E. Maggiolini , C. Gueli , N. Goshi , L. Fadiga , D. Ricci , S. Kassegne , T. Stieglitz , 2017, 4, 288. 10.1038/srep40332 PMC5234039 28084398

[87] L. Hiendlmeier , F. Zurita , J. Vogel , F. D. Duca , G. A. Boustani , H. Peng , I. Kopic , M. Nikić , T. Teshima , B. Wolfrum , Adv. Mater. 2023, 35, 2210206. 10.1002/adma.202210206 36594106

[88] A. S. Pranti , A. Schander , A. Bödecker , W. Lang , Sens. Actuators, B 2018, 275, 382.

[89] E. K. Brunton , B. Winther‐Jensen , C. Wang , E. B. Yan , S. Hagh Gooie , A. J. Lowery , R. Rajan , Front. Neurosci. 2015, 8, 5. 10.3389/fnins.2015.00265 PMC4518750 26283905

[90] D. K. Chelvanayagam , R. M. Vickery , M. T. K. Kirkcaldie , M. T. Coroneo , J. W. Morley , J. Neural Eng. 2008, 5, 125. 18382049 10.1088/1741-2560/5/2/003

[91] T. Allison‐Walker , M. A. Hagan , N. S. C. Price , Y. T. Wong , Brain Stimul. 2021, 14, 741. 33975054 10.1016/j.brs.2021.04.020

[92] A. Corna , T. Herrmann , G. Zeck , J. Neural Eng. 2018, 15, 045003. 29717707 10.1088/1741-2552/aac1c8

[93] H. W. Seo , N. Kim , J. Ahn , S. Cha , Y. S. Goo , S. Kim , J. Neural Eng. 2019, 16, 056016. 31357188 10.1088/1741-2552/ab36ab

[94] S. Staufert , H. Torlakcik , S. Pane , C. Hierold , IEEE Trans. Nanobiosci. 2019, 18, 230. 10.1109/TNB.2019.2905498 30892225

[95] Y.‐S. Sun , H.‐H. Huang , Y.‐H. Tsai , Y.‐L. Kuo , J.‐W. Lee , Y.‐J. Lee , T. Y. Linn , P. Chen , J. Dent. Sci. 2024, 19, S70. 39807433 10.1016/j.jds.2024.09.007 PMC11725070

[96] A. Belu , M. Yilmaz , E. Neumann , A. Offenhäusser , G. Demirel , D. Mayer , J. Biomed. Mater. Res., Part A 2018, 106, 1634. 10.1002/jbm.a.36363 29427541

[97] F. Milos , A. Belu , D. Mayer , V. Maybeck , A. Offenhäusser , Adv. Biol. 2021, 5, 2000248.

[98] T. Takekawa , Y. Isomura , T. Fukai , Eur. J. Neurosci. 2010, 31, 263. 20074217 10.1111/j.1460-9568.2009.07068.x

[99] K. Deligkaris , T. Bullmann , U. Frey , Front. Neurosci. 2016, 10, 421. 27683541 10.3389/fnins.2016.00421 PMC5021702

[100] K. Sakai , T. F. Teshima , T. Goto , H. Nakashima , M. Yamaguchi , Adv. Funct. Mater. 2023, 33, 2301836.

[101] J. R. Buitenweg , W. L. Rutten , W. P. Willems , J. W. van Nieuwkasteele , Med. Biol. Eng. Comput. 1998, 36, 630. 10367450 10.1007/BF02524436

[102] R. Matsumura , H. Yamamoto , M. Niwano , A. Hirano‐Iwata , Appl. Phys. Lett. 2016, 108, 023701. 27703279 10.1063/1.4939629 PMC5035130

[103] B. Miccoli , C. M. Lopez , E. Goikoetxea , J. Putzeys , M. Sekeri , O. Krylychkina , S.‐W. Chang , A. Firrincieli , A. Andrei , V. Reumers , D. Braeken , Front. Neurosci. 2019, 13. 10.3389/fnins.2019.00641 PMC6603149 31293372

[104] L. Wang , M. Riss , J. O. Buitrago , E. Claverol‐Tinturé , J. Neural Eng. 2012, 9, 026010. 22333069 10.1088/1741-2560/9/2/026010

[105] F. Santoro , S. Dasgupta , J. Schnitker , T. Auth , E. Neumann , G. Panaitov , G. Gompper , A. Offenhäusser , ACS Nano 2014, 8, 6713. 24963873 10.1021/nn500393p

[106] M. E. Spira , A. Hai , Nat. Nanotechnol. 2013, 8, 83. 23380931 10.1038/nnano.2012.265

[107] B. Hofmann , E. Kätelhön , M. Schottdorf , A. Offenhäusser , B. Wolfrum , Lab Chip 2011, 11, 1054. 21286648 10.1039/c0lc00582g

[108] J. Abu Shihada , M. Jung , S. Decke , L. Koschinski , S. Musall , V. Rincón Montes , A. Offenhäusser , Adv. Sci. 2024, 11, 2305944. 10.1002/advs.202305944 PMC10987114 38240370

[109] V. Viswam , M. E. J. Obien , F. Franke , U. Frey , A. Hierlemann , Front. Neurosci. 2019, 13. 10.3389/fnins.2019.00385 PMC6498989 31105515

[110] M. Kollo , R. Racz , M.‐E. Hanna , A. Obaid , M. R. Angle , W. Wray , Y. Kong , J. Müller , A. Hierlemann , N. A. Melosh , A. T. Schaefer , Front. Neurosci. 2020, 14. 10.3389/fnins.2020.00834 PMC7432274 32848584

[111] V. N. Vernekar , M. C. LaPlaca , Biomed. Eng. Lett. 2020, 10, 579. 33194249 10.1007/s13534-020-00166-5 PMC7655887

[112] P. Charlesworth , E. Cotterill , A. Morton , S. G. Grant , S. J. Eglen , Neural Dev. 2015, 10, 1. 25626996 10.1186/s13064-014-0028-0 PMC4320829

[113] M. Bisio , A. Bosca , V. Pasquale , L. Berdondini , M. Chiappalone , PLoS One 2014, 9, 107400. 10.1371/journal.pone.0107400 PMC4175468 25250616

[114] D. Lam , H. A. Enright , J. Cadena , S. K. G. Peters , A. P. Sales , J. J. Osburn , D. A. Soscia , K. S. Kulp , E. K. Wheeler , N. O. Fischer , Sci. Rep. 2019, 9, 4159. 30858401 10.1038/s41598-019-40128-1 PMC6411890

[115] M. Siebler , H. Köller , C. C. Stichel , H. W. Müller , H.‐J. Freund , Synapse 1993, 14, 206. 8211707 10.1002/syn.890140304

[116] E. Cotterill , D. Hall , K. Wallace , W. R. Mundy , S. J. Eglen , T. J. Shafer , SLAS Discovery 2016, 21, 510. 10.1177/1087057116640520 PMC4904353 27028607

[117] M. S. Schroeter , P. Charlesworth , M. G. Kitzbichler , O. Paulsen , E. T. Bullmore , J. Neurosci. 2015, 35, 5459. 25855164 10.1523/JNEUROSCI.4259-14.2015 PMC4388914

[118] K. Shimba , K. Sakai , Y. Takayama , K. Kotani , Y. Jimbo , Biomed. Microdevices 2015, 17, 94. 26303583 10.1007/s10544-015-9997-y

