# Supplementary material for: UV Laser‐Induced Carbon Microelectrode Arrays for Neuronal Recordings
Source: Adv Healthc Mater. 2025 Aug 20;14(30):e02136. doi: 10.1002/adhm.202502136 (PMC12645077; doi:10.1002/adhm.202502136)
Supplement: Supplementary file 1 — Supporting Information [file ADHM-14-0-s001.docx]

Supporting Information

UV Laser-Induced Carbon Microelectrode Arrays for Neuronal Recordings

Fulvia Del Duca^1,2^, Koji Sakai^2,3*^, Beatrice De Chiara^1^, Toichiro Goto^2,3^, Defne Tüzün^1^, Lukas Hiendlmeier^1^, George Al Boustani^1^, Hu Peng^1^, Tetsuhiko F. Teshima^1,4^, Simon N. Jacob^5^, and Bernhard Wolfrum^1,4*^

1 Neuroelectronics, Munich Institute of Biomedical Engineering, Department of Electrical Engineering, School of Computation, Information and Technology, Technical University of Munich, Hans-Piloty-Str. 1, 85748 Garching, Germany

2 Basic Research Laboratories, NTT Inc., 3-1 Morinosato Wakamiya, Atsugi, Kanagawa 243-0198, Japan

3 NTT Bio-Medical Informatics Research Center, NTT Inc., 3-1 Morinosato Wakamiya, Atsugi, Kanagawa 243-0198, Japan

4 Medical and Health Informatics Laboratories, NTT Research Incorporated 940 Stewart Dr, Sunnyvale, CA 94085, USA

5 Translational Neurotechnology Laboratory, Department of Neurosurgery, Klinikum rechts der Isar, Technical University of Munich, Munich, 81675, Germany

**Corresponding authors*


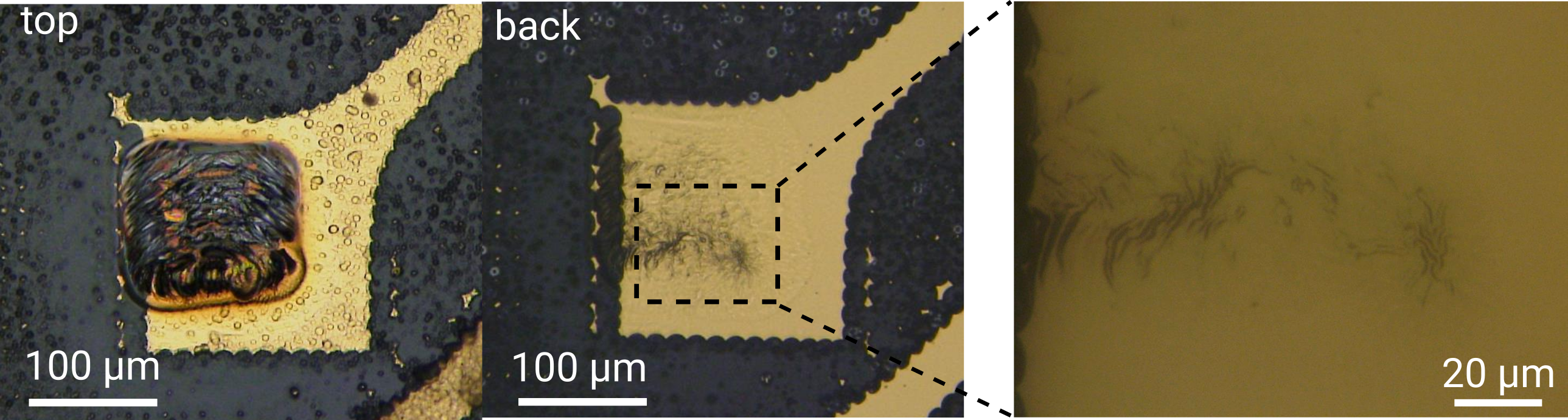
E-mail addresses: ko.sakai@ntt.com (K. Sakai), bernhard.wolfrum@tum.de (B. Wolfrum).

**Figure S1**. Optical microscopy images of an exemplary electrode from the top side and from the back side. The inset shows a zoom on the deformation of the gold layer underneath the carbonized parylene area.


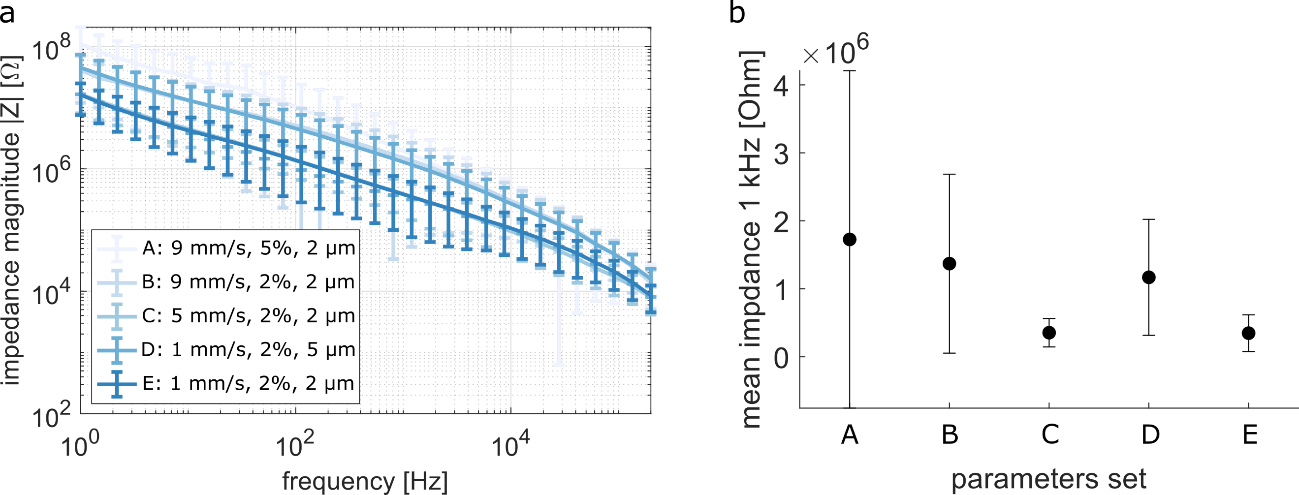


**Figure S2.** Impedance of electrodes fabricated at different laser parameters. (a) Impedance spectra of electrodes fabricated at different laser parameters (mean±SD, n = 6). (b) Impedance mean and standard deviatiation at 1 kHz (mean±SD, n = 6).


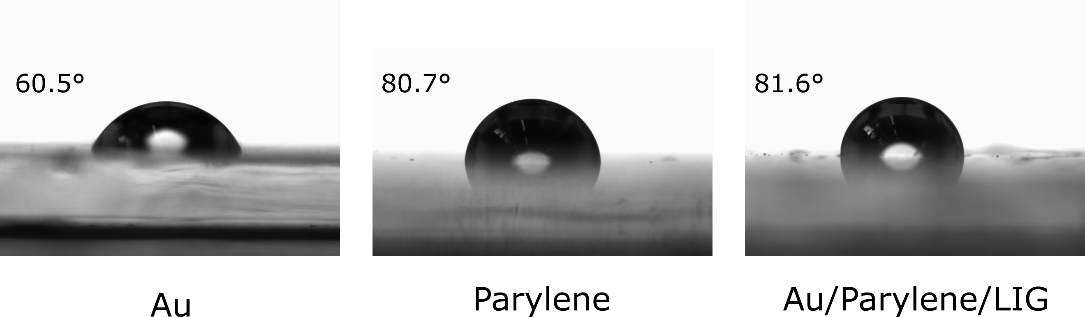


**Figure S3.** Optical contact angle measurements of Au, parylene, and the laser-induced carbon.


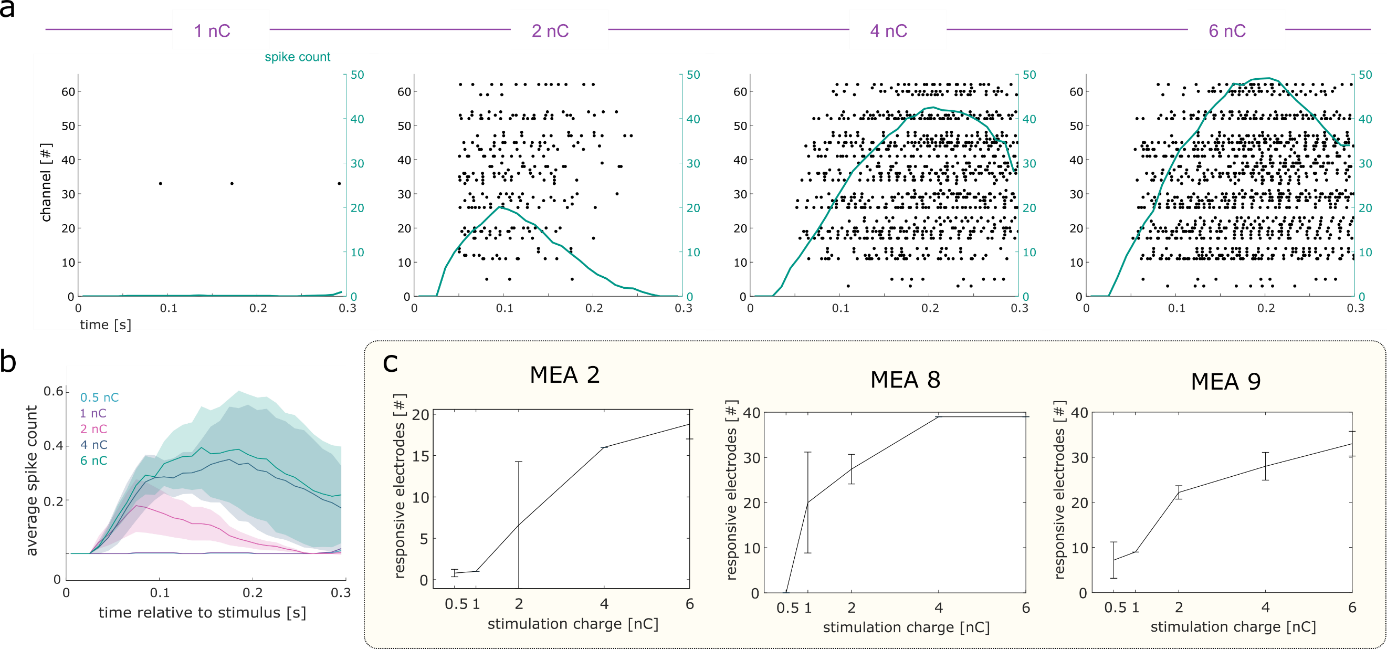


**Figure S4.** In vitro stimulation experiment of primary neuronal cultures. (a) Raster plot of one MEA, for stimulations at different charge per phase. (b) Average histograms and standard deviation of n = 5 stimulation trials. (c) Responsive electrodes per charge for the other 3 MEAs.


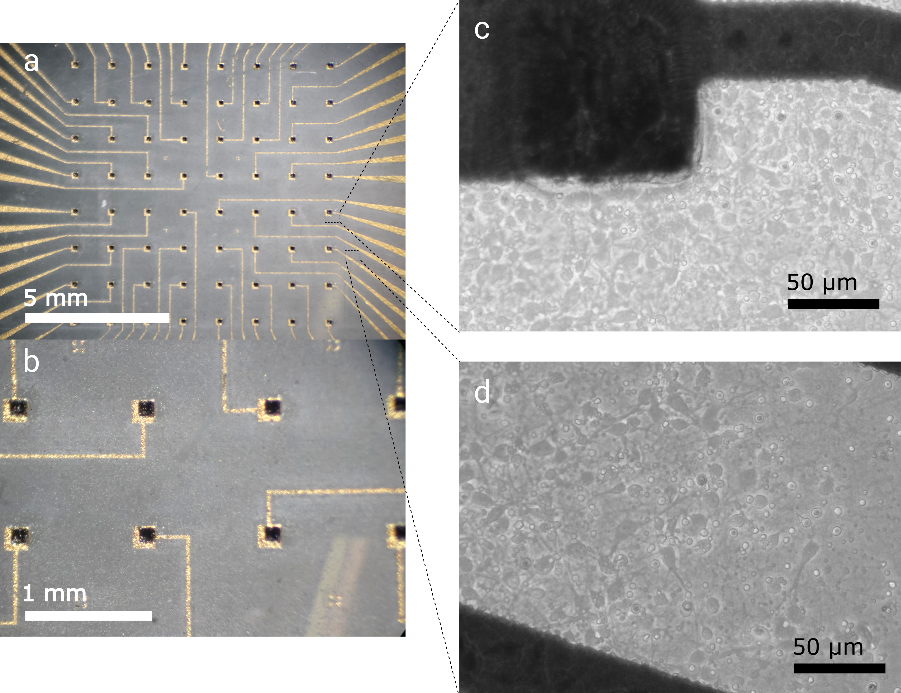


**Figure S5.** Microelectrode arrays used for in vitro experiments. (a, b) Optical microscopy images of one exemplary MEA, before culturing cells. (c, d) Phase-contrast images of MEAs cultured with primary hippocampal neurons at DIV 4.
